# Bibliographical

**Published:** 1873-02

**Authors:** 


### BIBLIOGRAPHICAL.

A SYSTEM OF ORAL SURGERY: Being a Consideration of the Diseases of Surgery of the Mouth, Jaws, and Associated Parts. By JAMES E. GARRETSON, M.D., D.D. S , Oral Surgeon to the Medical Department of the University of Pennsylvania, etc., etc. Illustrated with Numerous Steel Plates and Wood Cuts. Philadelphia: J. B. LIPPINCOTT & Co. 1873.

The author has, in the above work, given the practitioners of medicine and dentistry a volume so thorough and complete that each will find it indispensable. The author tells us he has "exerted himself to make it a system; step by step, from the elements of the subject—from the first departures from normal life—attempt has been made to follow the various conditions to and through their profoundest complications."

Few practitioners have taken the trouble to inform themselves as they should concerning the diseases of the mouth, jaws and associated parts; and in writing this system the author "has had continuously in mind the recognition of the important fact that in no department of medical science has there existed a hiatus such as that found to-day between general surgery and dentistry—a lacking span, truly, in the bridge of practice."

That the author has "bridged" this "span" to a very great degree, we most assuredly believe—for he has given a work so thorough in its details and so practical in its teachings as to bring before the profession at one view every disease and surgical operation for practical study and treatment. In doing this, no unnecessary ground has been wasted; and yet the volume is large, consisting of over one thousand pages, with fifty-one chapters.

In a work the scope of which is so extensive, it becomes impossible to enter into particulars and at the same time to do it justice. Under the head of associate lesions of first dentition, the author gives a group of diseases—diseases, he remarks, if they may be so called, directly associated with and dependent on abnormal dentition, and having, therefore, necessarily their cure more or less intimately associated with the correction of the primary lesion. These are: 1. Localized stomatitis; 2. Irritative fever; 3. Diarrhœa; 4. Spasms; 5. Eruptions of the skin—especially of the scalp and face. The pathology, causes, etc., of these diseases, with the treatment, are fully considered, and the practitioner will find this part of the work eminently practical and the treatment judicious.

The chapter on neuralgia is perhaps one of the best of the work—even where all are excellent—and the treatment recommended is as comprehensive and exhaustive as its known pathology and modern therapeutics can make it.

As before stated, the work is what it purports to be—a system—so complete and convenient that no practitioner should voluntarily deprive himself of it.

The plates and illustrations are numerous, adding much to the value of the work, while the letter press is in the very best style of the well-known publishers, J. B. Lippincott & Co.

A Practical Treatise on Urinary and Renal Diseases, Including Urinary Deposits. Illustrated by Numerous Cases and Engravings. By William Roberts, M.D., Fellow of the Royal College of Physicians, London, etc. Second American Edition, from the Second Revised and Considerably Enlarged London Edition. Philadelphia: Henry C. Lea. 1872.

The first edition of this fine work was exhaused three years ago, a fact which called for a second edition. While the plan of the work "remains unaltered, all the chapters have been carefully revised, and considerable additions have been made to several of them. Two entirely new articles have been inserted, namely, those on Suppression of Urine and Paroxysmal Hæmatinuria. Several of the old engravings have been replaced by better ones, and twenty new ones have been added."

This work of Dr. Roberts is unquestionably the best "practical treatise on urinary and renal diseases" in our language, and is just such a work as every practitioner should possess who is desirous of becoming practically acquainted with the diseases of which it treats. After the introductory chapter the author discusses the physical properties of the urine; chemical constituents of the urine and their variations—inorganic deposits; abnormal substances—organic deposits; diabetes insipidus; diabetes mellitus; gravel and calculus; chylous urine; congestion of the kidneys; Bright's disease; acute Bright's disease; chronic Bright's disease; suppuration in the kidneys; renal embolism; pyelitis and pyonephrosis; concretions in the kidneys; hydronephrosis; cysts and cystic degeneration of the kidneys; cancer of the kidney; benign growth in the kidney; tubercle; entozoa in the kidney; anomalies of position, form and number of the kidneys.

OBSTETRIC APHORISMS: For the use of Students Commencing Midwifery Practice. By JOSEPH GRIFFITHS SWAYNE, M.D., Physician Accoucheur to the Bristol General Hospital, etc., etc. Second American from the Fifth Revised English Edition, with Additions. By EDWARD R. HUTCHINS, M.D. Philadelphia: HENRY C. LEA. 1873.

This is an instructive little volume of one hundred and eighty-nine pages. The student just commencing obstetrical practice will find in this volume a trustworthy friend, while the general practitioner can glean from its pages many valuable thoughts for practical use at the bedside. Within the compass of one hundred and eighty-nine pages, we know of no work containing so many practical truths.

FACTS OF VITAL STATISTICS IN THE UNITED STATES: with Tables and Diagrams. Extracts from an Address. By J. M. TONER, M.D.

FREE PARKS AND CAMPING-GROUNDS: or Sanitariums for the Sick and Debilitated Children of large Cities during the Summer Months. By J. M. TONER, M.D., Washington City, D.C.

A PAPER: on the Propriety and Necessity of Compulsory Vaccination. By J. M. TONER, M.D.

These pamphlets of Dr. Toner are valuable contributions to the medical and statistical literature of the country, and should have a large reading by the profession. At some future time we will give extracts from them for the use of our readers.

NEW TREATMENT OF VENEREAL DISEASES AND OF ULCERATIVE SYPHILITIC AFFECTIONS BY IODOFORM. Translated from the French of Dr. A. A. IZARD. By HOWARD F. DAMEN, M.D. Boston: JAMES CAMPBELL. Price, Fifty Cents.

This is a very readable and instructive volume of seventy-three pages, and so cheap that every one may secure a copy.

THE USE OF THE SETON IN THE TREATMENT OF SOME CASES OF CHRONIC AFFECTIONS OF THE WOMB. By ELY VAN DE WARKER, M.D., Physician for Diseases of Women to the Syracuse Free Dispensary. New York: J. Ross & Co., Printers, 27 Rose Street.

Everything from the pen of Dr. Ely Van De Warker has the impress of earnest and scientific thought. The employment of the seton by the Doctor in the cases given seems to have shortened the usual tedious treatment, and opens up a new method—and we trust a safe and expeditious one—for the speedy cure of hypertrophied conditions of the cervix uteri. His treatment is worth repeating, and the profession would do well to bear it in mind when suitable cases present themselves.

BURNS AND SCALDS: their Treatment, with Cases. Read before the Sacramento Society for Medical Improvement. By JOS. F. MONTGOMERY, M.D.

An Examination of Professor Reese's "Review of the Trial of Mrs. Wharton for the Murder of General Ketchum." By Philip C. Williams, M.D., of Baltimore, Maryland.

Annual Report of the Trustees and Superintendent of the Mississippi State Lunatic Asylum for the Year 1872.

The management of this institution is highly creditable to the Superintendent, William M. Compton, M.D., as evined by the report before us.

Sudden Death in Puerperal Cases. By S. L. Jepson, M.D., of Wheeling, West-Virginia.

Dr. Jepson has written the best monograph upon "Sudden Death in Puerperal Cases" it has been our pleasure to read. His pamphlet should be in the hands of all practitioners.

An Address before the Waco Medical Association. By D. R. Wallace, A.M., M.D.

This address has the "ring of the true metal," and speaks highly for the learning and ability of our brethren of Texas.

